# Bioactive oral gels containing *Libidibia ferrea* and Brazilian propolis preserve the mechanical and mineral properties of eroded human enamel

**DOI:** 10.1007/s00784-026-07215-w

**Published:** 2026-10-13

**Authors:** Diana Moreira Gomes Martins, Alexia Vaughan Paz, Rafael Bestene de Oliveira dos Santos, Mariana Tenório Picanço, Ricardo Roberto de Souza Fonseca, Milton Carlos Kuga, Mileide da Paz Brito, Cristiane de Melo Alencar

**Affiliations:** 1 School of Dentistry, University Center of State of Pará (CESUPA), 9th of january street, 927, São Braz, 66060-575 Belém, Pará Brazil; 2 School of Dentistry, São Paulo State University (UNESP), Araraquara, São Paulo, Brazil

**Keywords:** Dental erosion, Enamel, Polyphenols, Raman spectroscopy, Antioxidants

## Abstract

**Objectives:**

To develop polyphenol-rich oral gels containing *Libidibia ferrea* or Brazilian brown propolis extracts and evaluate their effects on the mechanical and mineral properties of human enamel subjected to erosive–abrasive challenges.

**Materials and methods:**

Experimental gels containing *L. ferrea* (0.5% and 2.5%) or Brazilian brown propolis (0.5% and 2.5%) were formulated and evaluated for physicochemical properties, antioxidant activity, stability, and cytocompatibility. Seventy-two human enamel specimens were randomly assigned to six groups (*n* = 12): control gel, 0.5% and 2.5% *L. ferrea* gels, 0.5% and 2.5% propolis gels, and a commercial stannous fluoride toothpaste. Specimens underwent a 7-day erosive–abrasive cycling model. Surface microhardness, surface roughness, enamel crystallinity, phosphate-to-carbonate ratio, and color variation were assessed. Data were analyzed using ANOVA and Tukey’s test (α = 0.05).

**Results:**

All formulations exhibited near-neutral pH and satisfactory cytocompatibility. Stability was maintained under refrigeration, whereas color changes and precipitate formation occurred during storage at room and elevated temperatures. Following the erosive–abrasive challenge, the 2.5% *L. ferrea* and 2.5% propolis gel groups showed higher final surface microhardness, lower percentage surface microhardness loss, and lower final surface roughness than the other treatment groups (*p* < 0.05). Raman spectroscopy showed lower full width at half maximum values for the phosphate ν₁ band and higher phosphate-to-carbonate ratios in the 2.5% *L. ferrea* and 2.5% propolis gel groups than in the other groups (*p* < 0.05).

**Conclusions:**

Oral gels containing 2.5% *L. ferrea* or Brazilian brown propolis showed promising protective effects on human enamel under the in vitro erosive–abrasive conditions tested.

**Clinical Relevance:**

These gels are promising candidates for erosive tooth wear prevention, pending in situ and clinical validation addressing color changes and concentration-dependent stability.

## Introduction

Erosive tooth wear is a multifactorial process involving the cumulative, irreversible loss of dental hard tissues through the interaction of chemical and mechanical factors, without bacterial involvement [1]. Over the past decades, its prevalence has increased substantially across different age groups, becoming an important concern for preventive and restorative dentistry [2, 3]. Dental erosion, the chemical component of this process, involves acid-induced dissolution of enamel minerals, leading to surface softening and changes in surface characteristics and mineral organization [4]. The softened enamel surface becomes more susceptible to mechanical wear, including toothbrushing abrasion, which can accelerate tissue loss and exacerbate the clinical consequences of erosive tooth wear [5]. Therefore, the development of preventive strategies capable of minimizing mineral loss and preserving enamel structural integrity under erosive–abrasive conditions remains a major challenge in contemporary dentistry.

Fluoride-containing products are widely investigated for the prevention of erosive tooth wear, including formulations containing sodium fluoride or stannous fluoride [6–8]. Their protective effects vary according to the fluoride compound, formulation, concentration, exposure conditions, and associated mechanical abrasion [6–9]. These factors may limit protection under certain erosive–abrasive conditions, supporting the investigation of complementary bioactive approaches that may enhance surface resistance or modify interactions between dental tissues and the acquired salivary pellicle [10].

Natural products rich in polyphenols have attracted increasing attention in this context due to their antioxidant, anti-inflammatory, and biomodulatory properties [11, 12]. In dentistry, polyphenolic compounds have been investigated for their ability to interact with extracellular matrix proteins, inhibit enzymatic degradation, and modify mineralized tissue surfaces [13, 14]. Recent evidence suggests that these compounds may contribute to the preservation of dental tissues by reducing mineral dissolution, enhancing surface stability, and modulating physicochemical processes involved in erosion [15, 16]. Furthermore, antioxidant activity has been suggested as a potentially relevant property of polyphenol-rich compounds investigated for dental erosion prevention [17, 18], although its specific contribution to enamel protection remains unclear.

Among the Brazilian bioresources with high polyphenol content, Libidibia ferrea (jucá) and Brazilian propolis stand out because of their rich composition of phenolic acids, flavonoids, tannins, and other bioactive compounds [19, 20]. Previous studies have demonstrated their antioxidant, antimicrobial, anti-inflammatory, and tissue-protective properties [21–24]. However, despite the growing interest in these natural products, limited information is available regarding their incorporation into oral gel formulations and their potential role in protecting enamel against erosive tooth wear under erosive–abrasive conditions. The present study addresses this gap by evaluating gels containing two concentrations of each extract through complementary assessments of enamel mechanical, surface, and Raman-derived mineral parameters, alongside formulation characterization.

It remains unclear whether oral gels containing *Libidibia ferrea* or Brazilian brown propolis can limit changes in enamel mechanical, surface, and mineral parameters under erosive–abrasive conditions, and whether these effects differ according to extract concentration. A Carbopol-based vehicle was selected to facilitate topical application, while concentrations of 0.5% and 2.5% were chosen on an exploratory basis to investigate enamel effects at relatively low extract loadings while limiting potential discoloration. Therefore, this study aimed to develop and characterize these formulations and evaluate their effects on human enamel subjected to erosive–abrasive challenges. The null hypothesis was that enamel surface microhardness, roughness, phosphate-band full width at half maximum, and phosphate-to-carbonate ratio would not differ among treatment groups following the erosive–abrasive challenge.

## Materials and methods

### Preparation of experimental gels

Brazilian brown propolis and *Libidibia ferrea* (jucá) pods were obtained in Pará State, in the Brazilian Amazon. Libidibia ferrea (jucá) pods were obtained from Acará, Pará State, in the Brazilian Amazon, and their botanical identity was confirmed by a botanist. Raw propolis produced by Apis mellifera was obtained from Ouremmel, Pará, Brazil, and classified as Brazilian brown propolis based on its coloration and identification by the supplier’s botanist. Its specific botanical source was not confirmed. Access to Brazilian genetic heritage was registered in the National System for the Management of Genetic Heritage and Associated Traditional Knowledge (SisGen) under registration numbers AD49477 and ACDE09A.

Jucá pods and raw Brazilian brown propolis resin (1000 g each) were washed under running water, rinsed with distilled water, and immersed in boiling distilled water for 5 min to remove surface contaminants. The materials were subsequently dried in a forced-air circulation oven (Orion 520, Fanem^®^, São Paulo, Brazil) at 47 °C for four days and ground into a fine powder using an industrial blender (KD Eletro^®^, São Paulo, Brazil).

Hydroalcoholic extracts (HE) were obtained by macerating the powdered materials in 1000 mL of an ethanol–water solution (70:30, v/v) for five days at room temperature under continuous stirring. The extracts were filtered and concentrated under reduced pressure in a rotary evaporator (Büchi^®^, Flawil, Switzerland) at 30 °C. The concentrated material was subsequently freeze-dried (Christ^®^, Osterode am Harz, Germany) for 72 h at − 101 °C and 23mmHg. The resulting dried extracts (50 g) were stored at − 20 °C until use.

Experimental gels were prepared using Carbopol^®^ 940 (2%), propylene glycol (5%), methylparaben (0.2%), ethylenediaminetetraacetic acid (EDTA, 0.1%), purified water (q.s.), and hydroalcoholic extracts of Brazilian brown propolis or *Libidibia ferrea* at concentrations of 0.5% and 2.5%. All ingredients were weighed using an analytical balance (Shimadzu, Barueri, SP, Brazil) and dispersed in purified water under continuous stirring until complete homogenization. The gel base was neutralized with 50% triethanolamine solution (q.s.) to obtain the desired consistency and pH, following the recommendations of the Brazilian National Formulary [25]. After complete gel formation, the plant extracts were incorporated into the Carbopol^®^ matrix at the respective concentrations. The formulations were homogenized and packaged after reaching room temperature.

### Chemical characterization of the experimental gels

The pH of the gel formulations was measured using a digital pH meter (W3B, BEL Engineering^®^, Monza, Italy), previously calibrated with standard buffer solutions at pH 4.0 and 7.0 according to the manufacturer’s instructions. Measurements were performed at room temperature (approximately 25 °C), and the mean values were recorded for each formulation.

Total phenolic content (TPC) was determined using the Folin–Ciocalteu colorimetric method, adapted from Waterhouse (2002) [26]. Briefly, 100 mg of each gel formulation (in triplicate) was mixed with 7mL of deionized water and 500 µL of Folin–Ciocalteu reagent. After 1–8 min, 1.5mL of 20% sodium carbonate was added, and the mixtures were incubated in the dark for 2 h. Absorbance was measured at 765 nm using a UV–Vis spectrophotometer (Agilent Cary 60, USA). A gallic acid calibration curve (10–500 µg/mL⁻¹) was used, and the results were expressed as micrograms of gallic acid equivalents per milligram of gel (µg GAE mg⁻¹ gel).

Antioxidant capacity was assessed using DPPH and ABTS radical-scavenging assays according to Rufino et al. [27], with minor modifications. Both assays were performed in 96-well flat-bottom microplates (Nunc MicroWell, Nunclon Delta-treated, Thermo Fisher Scientific, Waltham, MA, USA). Gel samples were dispersed in HPLC-grade methanol at 500 µg mL⁻¹ and serially diluted to concentrations ranging from 500 to 7.81 µg mL⁻¹.

For the DPPH assay, 100 µL of each dilution was mixed with 40 µL of a 0.3 mM DPPH solution in methanol. Methanol containing DPPH served as the negative control, and Trolox [(±)-6-hydroxy-2,5,7,8-tetramethylchromane-2-carboxylic acid] was used as the positive control and reference standard at concentrations ranging from 0.78 to 50 µg mL⁻¹. Reaction mixtures were incubated in the dark for 30 min at room temperature, and absorbance was measured at 492 nm using a microplate spectrophotometer. Results were expressed as EC₅₀, defined as the mass of gel required to reduce the initial DPPH radical amount by 50%, expressed in grams of gel per gram of DPPH (g/g DPPH).

For the ABTS assay, 20 µL of each dilution was mixed with 280 µL of freshly prepared ABTS radical cation (ABTS•⁺) solution. The negative control consisted of 20 µL of methanol and 280 µL of ABTS•⁺ solution. A Trolox calibration curve was prepared at concentrations ranging from 50 to 500 µM. Reaction mixtures were incubated in the dark for 20 min at room temperature, and absorbance was measured at 734 nm using a microplate spectrophotometer. Results were expressed as micromoles of Trolox equivalents per gram of gel (µmol TE g⁻¹). All reactions in both assays were performed in triplicate.

### Physical characterization of the experimental gels

The rheological properties of the experimental gel formulations were investigated using a controlled-stress rheometer (Physica MCR 301, Anton Paar, Ostfildern, Germany) fitted with a stainless-steel cone-and-plate measuring system (50 mm diameter, 2° cone angle, and 208 μm measuring gap). Analyses were carried out at a controlled temperature of 25 °C.

Flow behavior was assessed by subjecting the samples to shear rates ranging from 0 to 150s⁻¹, with 100 measurement points collected throughout each test. The resulting flow curves were generated from the relationship between shear rate and shear stress, allowing the determination of the viscosity profile and rheological characteristics of the formulations. All experiments were performed in triplicate to ensure reproducibility of the measurements [28].

For preliminary stability testing, 5 g of each formulation was distributed into glass vials and stored under four conditions: refrigeration (5 ± 2 °C), room temperature (27 °C), elevated temperature (40 ± 2 °C), and freeze–thaw cycling (alternating 24 h at 5 ± 2 °C and 24 h at 40 ± 2 °C for six cycles). Samples were evaluated at predetermined intervals for signs of physical instability. Accelerated stability was assessed over 90 days under refrigeration (5 ± 2 °C), room temperature (27 °C), and elevated temperature (40 ± 2 °C). Evaluations were performed at T0, T7, T15, T30, T60, and T90, including pH, appearance, color, odor, homogeneity, brightness, and the presence of precipitates or clumps [29].

### Biological characterization of the experimental gels

The cytocompatibility of the gel formulations was evaluated using immortalized human gingival fibroblasts (HGF-1). Cells were cultured in DMEM supplemented with 10% fetal bovine serum and maintained at 37 °C in a humidified atmosphere containing 5% CO₂. After reaching approximately 80% confluence, cells were seeded into 24-well plates at a density of 1 × 10⁵ cells/mL and exposed to the experimental gels for 24 h.

Cell viability was determined using the MTT assay (0.5 mg/mL). Following 3 h of incubation, formazan crystals were solubilized with 200µL of dimethyl sulfoxide (DMSO), and absorbance was measured at 570 nm using a microplate reader (BIO-RAD Model 450, São Paulo, Brazil). Cells treated with 10% formalin served as the non-viable control, whereas untreated cells were used as the viable control. Results were expressed as optical density (OD570). All experiments were performed in triplicate and repeated independently three times [30].

### Ethical considerations and sample size

All experimental procedures involving human biological materials were carried out in accordance with the ethical standards established by the Declaration of Helsinki and adhered to the recommendations of the International Committee of Medical Journal Editors (ICMJE). Human enamel specimens were prepared from extracted third molars donated by volunteers after providing written informed consent. Saliva samples were collected from healthy adult participants under the same consent procedures.

All protocols involving the collection, handling, and use of human tissues complied with applicable Brazilian National Health Council Resolutions No. 466/2012 and 510/2016 governing human research. It was approved by the Research Ethics Committee under protocol number 8.515.751.

The required sample size was estimated using G*Power software (Heinrich Heine University Düsseldorf, Düsseldorf, Germany), based on dentin surface loss data from a previous in vitro study evaluating cocoa antioxidant extract in different delivery vehicles [16]. For a one-way ANOVA with six groups, an estimated effect size of Cohen’s f ≈ 0.44, a significance level of 5% (α = 0.05), and 80% statistical power indicated a minimum total sample of 72 specimens (12 per group).

### Study protocol

#### Saliva collection

Whole saliva was collected from 20 healthy volunteers (11 males and 9 females; 18–28 years old). Individuals who were smokers, pregnant, taking continuous medication, or diagnosed with systemic diseases were excluded. Additional exclusion criteria included active caries, erosive tooth wear, and periodontal disease. Only participants with normal salivary function were enrolled, defined by stimulated and unstimulated salivary flow rates greater than 1.0 mL/min and 0.3mL/min, respectively. Saliva samples were pooled and centrifuged at 14,000×g for 20 min at 4 °C. The resulting supernatant was collected, aliquoted, and stored at − 80 °C until use [31].

#### Enamel specimen preparation

Extracted human third molars were stored in 0.1% thymol solution at room temperature until use. A total of 100 enamel specimens (3 × 3 mm) were prepared from the central coronal region of the teeth. The crowns were sectioned at the cementoenamel junction using a precision cutting machine (Minitom, Struers S.A.S., Champigny-sur-Marne, France) equipped with a double-sided diamond disc operating at low speed. To standardize specimen dimensions, two parallel diamond discs (Extec Corp., Lake Bluff, IL, USA) separated by a 2-mm spacer were used. All cutting procedures were performed under continuous deionized water irrigation to prevent overheating and structural damage.

The enamel blocks were sequentially polished with #600 and #1200 silicon carbide abrasive papers under running water, producing flat and standardized surfaces. Specimens were then ultrasonically cleaned in deionized water for 5 min to remove residual debris. Finally, the specimens were stored in deionized water in sealed containers and kept refrigerated at approximately 4 °C until use to prevent dehydration [31].

#### Specimen selection and baseline surface microhardness assessment

Baseline surface microhardness was evaluated using a microhardness tester fitted with a Knoop indenter (HM-200, Mitutoyo, Tokyo, Japan), applying a static load of 50 g for 5 s [15]. Five Knoop indentations were performed in the central region of each specimen, with a spacing of 100 μm between adjacent indentations. The mean of these five measurements was used as the baseline microhardness value for each specimen.

Before randomization, specimens were screened using a predefined eligibility criterion: baseline microhardness within ± 10% of the overall mean calculated from the individual mean values of all 100 specimens. Twenty-eight specimens fell outside this range and were excluded. The remaining 72 specimens were assigned identification numbers and randomly allocated to six experimental groups (*n* = 12). Data normality was assessed using the Shapiro–Wilk test in SPSS software (version 13.0). One-way ANOVA detected no statistically significant differences in baseline microhardness among groups (*p* > 0.05).

#### Experimental design and erosive–abrasive protocol

Seventy-two enamel specimens were randomly assigned to six experimental conditions (*n* = 12): (G1) Carbopol gel without active compounds; (G2) 2.5% *Libidibia ferrea* gel; (G3) 0.5% *Libidibia ferrea* gel; (G4) 2.5% Brazilian brown propolis gel; (G5) 0.5% Brazilian brown propolis gel; and (G6) a commercial anti-erosive toothpaste containing 0.454% stannous fluoride (SnF₂; approximately 1100ppm F⁻) (Oral-B Pro-Expert/Pro-Repair Anti-Erosion, Procter & Gamble, UK/EU).

The erosive–abrasive cycling model was conducted over seven consecutive days. At the beginning of each day, specimens were positioned in 24-well plates, and 1 µL of the assigned treatment was applied directly to each enamel surface using a single-channel micropipette (Lambda^®^ Plus, Corning^®^, Sigma-Aldrich, Darmstadt, Germany). For the positive control, the toothpaste was prepared as a 1:3 slurry before application. Treatments were applied once daily and remained in contact with enamel for 10 min. Specimens were then rinsed with deionized water for 3 s and transferred to 1 mL of freshly thawed human saliva supplemented with protease inhibitors for 60 min at 37 °C to allow acquired salivary pellicle formation.

Each experimental day comprised three erosive–abrasive episodes. Each episode consisted of immersion in 1% citric acid (pH 3.6; 30 mL per specimen) for 90 s at 25 °C without agitation, immediately followed by mechanical brushing using an automated brushing machine (Odeme^®^, São Carlos, Brazil). An individual ultra-soft toothbrush (Curaprox^®^ 5460, Kriens, Switzerland) was assigned to each specimen. Brushing was performed without dentifrice at 37 °C, at 150 cycles/min for 15 s under a 2 N load [12].

After each brushing procedure, specimens were rinsed with deionized water for 5 s and transferred to human saliva (pH 6.8; 30 mL per specimen) at 37 °C. Specimens remained in saliva for 2 h between successive erosive–abrasive episodes and for 16 h overnight after the third daily episode. The treatment and cycling procedures were adapted from Alencar et al. [32].

#### Surface microhardness (KHN) and roughness assessment (Ra)

Surface microhardness (KHN) and roughness (Ra) were assessed in all 12 specimens per group (72 specimens in total) at baseline (T0) and after completion of erosive–abrasive cycling (T1). KHN was measured using the microhardness tester described in Sect.  2.6.3, under a static load of 50 g for 5 s [15]. Five indentations, spaced 100 μm apart, were made in the central region of each specimen and averaged to obtain its KHN value at each evaluation time.

Ra was measured using a profilometer (SJ-201P, Mitutoyo, Kawasaki, Japan), calibrated before analysis, with a cutoff of 0.25 mm. Three readings were obtained at different locations on each enamel surface, spaced 0.5 mm apart. Ra represented the arithmetic mean of the absolute profile deviations from the centerline, and the three readings were averaged to obtain each specimen’s value.

#### Raman spectroscopy analysis

Raman spectroscopy was performed on all 12 specimens per group using a spectrometer (iHR320, Horiba Scientific, Kyoto, Japan) equipped with a 785-nm laser operating at 10 mW and a spectral resolution of 5 cm⁻¹. Each specimen was positioned perpendicular to the incident laser beam to standardize spectral acquisition.

Spectra were processed using OriginPro software (version 8.5, OriginLab Corp., Northampton, MA, USA), and Raman bands were fitted with Lorentzian functions. The full width at half maximum (FWHM) of the phosphate ν₁ band centered at approximately 960 cm⁻¹ was determined as an indirect indicator of enamel crystal organization [33]. The phosphate-to-carbonate ratio was calculated from the integrated areas of the phosphate band (960 cm⁻¹) and carbonate band (1070–1100 cm⁻¹), providing an indirect measure of relative phosphate and carbonate contributions to the Raman spectra [34].

#### Color evaluation

Color measurements were obtained from all 12 specimens per group using a spectrophotometer (VITA Easyshade, Vident, Brea, CA, USA). Readings were performed at the center of each specimen and repeated three times at each evaluation period. To minimize the influence of external light reflection, specimens were positioned against a dark background during all measurements. The spectrophotometer was calibrated with a white reflectance standard according to the manufacturer’s recommendations before each assessment. Color coordinates were recorded using the CIE L*a*b* system, where L* represents lightness (0–100), a* the red–green axis, and b* the yellow–blue axis. Mean values were calculated and used to determine color differences (ΔE₀₀) between baseline (T0) and post-treatment (T1) measurements according to the CIEDE2000 formula: ΔE₀₀ = [(ΔL′/kLSL)² + (ΔC′/kCSC)² + (ΔH′/kHSH)² + RT(ΔC′/kCSC)(ΔH′/kHSH)]^1/2^, where ΔL′, ΔC′, and ΔH′ correspond to differences in lightness, chroma, and hue, respectively.

### Statistical analysis

Statistical analyses were performed using IBM SPSS Statistics, Version 30.0 (IBM Corp., Armonk, NY, USA). Normality and homogeneity of variances were assessed using Shapiro–Wilk and Levene tests, respectively. KHN and percentage surface microhardness loss (%SHL) were the prespecified primary outcomes; Ra, Raman parameters, and color variation were secondary enamel outcomes.

KHN and Ra were analyzed using two-way mixed-design ANOVA, with treatment as the between-specimen factor and time (T0 and T1) as the within-specimen repeated factor, assessing main and interaction effects. %SHL, phosphate-band FWHM, phosphate-to-carbonate ratio, and color variation (ΔE00) were compared among groups using one-way ANOVA. Tukey’s post hoc comparisons were performed when appropriate. Each specimen was the experimental unit (*n* = 12 per group), with replicate readings averaged before analysis.

Formulation characterization outcomes—TPC, antioxidant activity (DPPH and ABTS), pH, rheological parameters, and cell viability—were analyzed using one-way ANOVA followed by Tukey’s test. Data are presented as mean ± SD, with α = 0.05.

Exploratory Pearson correlations and simple linear regressions assessed associations between ABTS activity and final Ra or %SHL using means from the four extract-containing gels (*n* = 4 formulations), excluding controls. These analyses were hypothesis-generating and did not establish causality or a mechanism of enamel protection.

## Results

### Chemical characterization of the experimental gels

The pH of the experimental formulations remained within a near-neutral range, varying from 6.91 to 7.43. The 2.5% *Libidibia ferrea* gel exhibited the lowest pH value (6.91), followed by the 0.5% Brazilian brown propolis gel (7.01), the 0.5% *Libidibia ferrea* gel (7.20), and the 2.5% Brazilian brown propolis gel (7.43).

TPC and antioxidant activity data are summarized in Table 1. The formulations containing *Libidibia ferrea*, regardless of concentration, exhibited higher TPC values than the corresponding propolis-based gels. A similar trend was observed for antioxidant activity. The 2.5% *Libidibia ferrea* formulation showed the strongest antioxidant performance, as evidenced by the lowest DPPH EC₅₀ value and the highest ABTS radical-scavenging activity. The 0.5% *Libidibia ferrea* gel ranked second, whereas both Brazilian brown propolis formulations displayed comparatively lower antioxidant responses.

**Table 1:** TPC and DPPH and ABTS assays of the experimental gel formulations

Gels	Extractable polyphenols (mg GAE/100 g)	DPPH EC50 (g/g DPPH)^b^	ABTS^+^ (µmol trolox/g)
2.5% *Libidibia ferrea* gel	3103.1± 54.9	433.4 ± 12.8	181.1 ± 7.3
0.5% *Libidibia ferrea* gel;	1083.1 ± 18.2	5431.4 ± 117.5	19.1 ± 1.8
2.5% Brazilian brown propolis gel	1192.1 ± 37.1	862.9 ± 19.1	74.2 ± 14.9
0.5% Brazilian brown propolis gel	634.2 ± 16.4	1104.7 ± 91.2	63.1 ± 2.9

^B^Concentration of antioxidant required to reduce the initial number of free radicals by 50%

### Physical characterization of the experimental gels

**Fig. 1:**
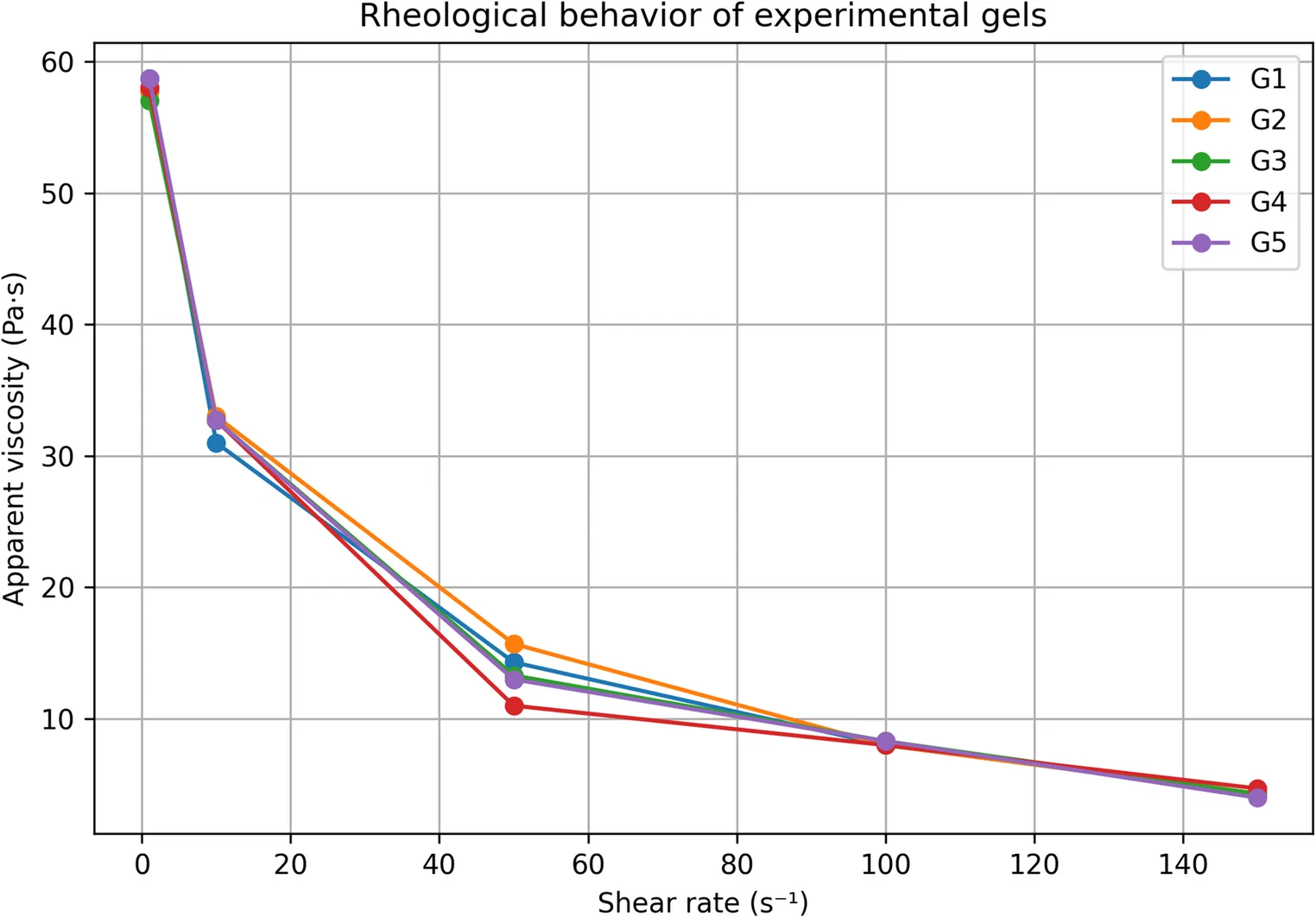
Rheological profiles of the experimental gel formulations. Apparent viscosity decreased as the shear rate increased, confirming the shear-thinning behavior characteristic of Carbopol-based gels

All formulations exhibited a marked decrease in apparent viscosity as the shear rate increased, demonstrating a typical non-Newtonian shear-thinning behavior. At the lowest shear rate (1s⁻¹), viscosity values ranged from 57.0 to 58.7 Pa·s, whereas at 150s⁻¹ they decreased to values between 4.0 and 4.7 Pa·s. No substantial differences were observed among the formulations, indicating that the incorporation of *Libidibia ferrea* and Brazilian brown propolis extracts did not significantly affect the rheological behavior of the Carbopol-based gel matrix (Fig. 1). The formulations remained stable under refrigeration throughout the study period, whereas color changes and precipitate formation were observed after prolonged storage at room temperature and under accelerated conditions (Table 2).

**Table 2:** Organoleptic stability of the experimental gel formulations under different storage conditions

Storage condition	T0	T7	T15	T30	T60	T90
Refrigeration	Stable^*^	Stable^*^	Stable^*^	Stable^*^	Stable^*^	Stable^*^
Room temperature	Stable^*^	Stable^*^	Stable^*^	Stable^*^	Stable^*^	Color change and precipitate formation
Elevated temperature	Stable^*^	Stable^*^	Color change and precipitate formation	Color change and precipitate formation	Color change and precipitate formation	Color change and precipitate formation
Freeze–thaw cycling	Stable^*^	Stable^*^ (Cycle 1)	Stable^*^ (Cycle 4)	Stable^*^ (Cycle 6)	–	–

^*^Stable = no changes in appearance, odor, homogeneity, or precipitate formation

### Cytocompatibility of the experimental gels

**Fig. 2:**
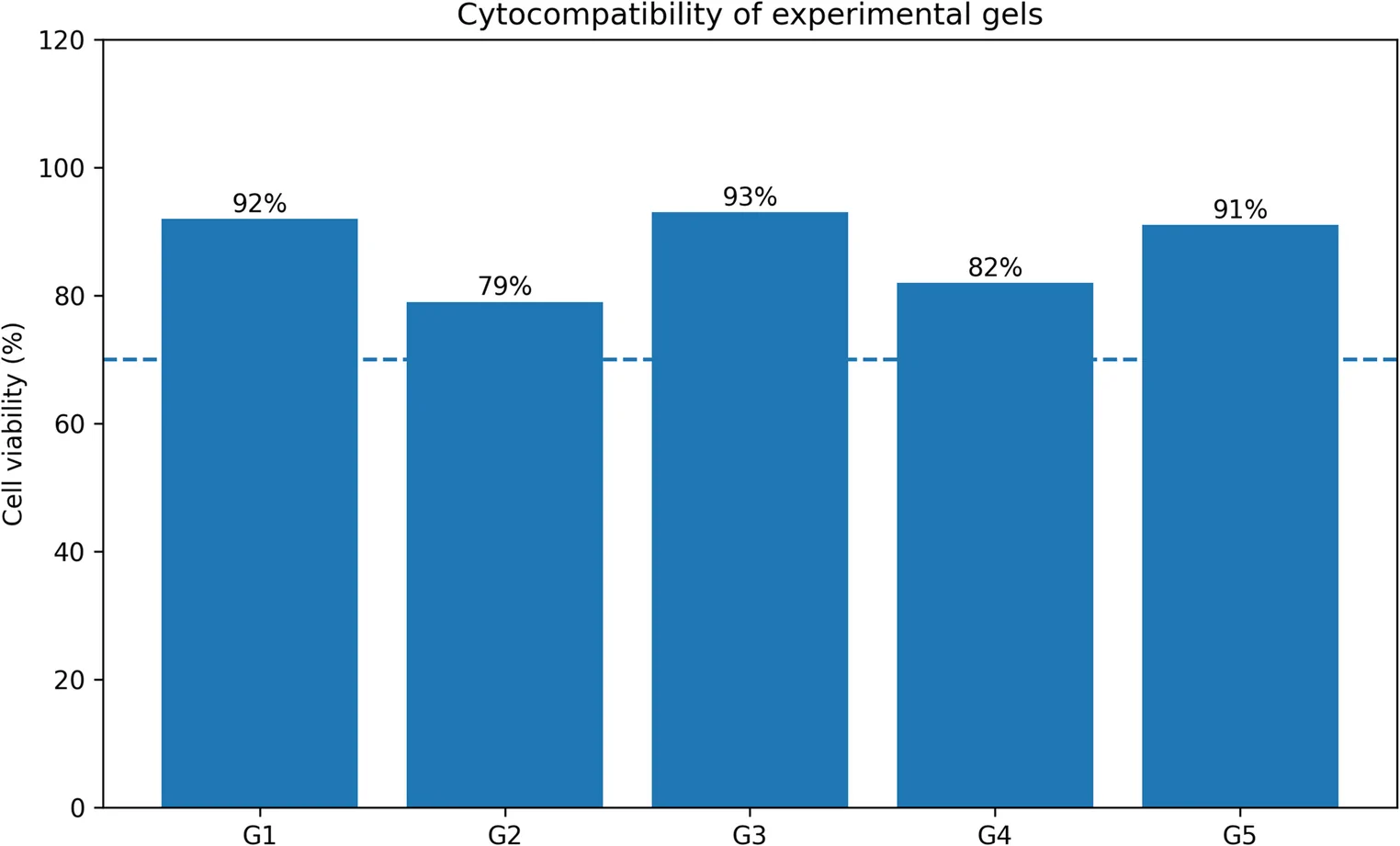
Cell viability of human gingival fibroblasts exposed to the experimental gel formulations. The dashed horizontal line represents the 70% viability threshold established by ISO 10993-5 for classification of non-cytotoxic materials

The cytocompatibility results are shown in Fig. 2. All formulations exhibited cell viability values above the 70% threshold established by ISO 10993-5, indicating the absence of cytotoxic effects. Cell viability ranged from 79% to 93%, with the highest value observed for the 0.5% *Libidibia ferrea* gel (G3; 93%) and the lowest for the 2.5% *Libidibia ferrea* gel (G2; 79%). The Carbopol control (G1), 0.5% Brazilian brown propolis gel (G5), and 2.5% Brazilian brown propolis gel (G4) exhibited viability values of 92%, 91%, and 82%, respectively. Overall, the incorporation of the plant extracts did not adversely affect cell viability, demonstrating satisfactory cytocompatibility of the experimental formulations.\

### KHN and Ra analysis

The KHN and %SHL are presented in Table 3. At baseline (T0), surface microhardness values showed no significant differences among groups (*p* = 0.075). Following the erosive–abrasive challenge (T1), all groups exhibited a significant reduction in surface microhardness compared to their respective baseline values (*p* < 0.05). However, significant differences were observed among treatments at T1. The highest final microhardness values were recorded for G2 and G4 (*p* < 0.05), which did not differ statistically from each other and displayed significantly higher values than G1, G3, G5, and G6 (*p* < 0.05). Conversely, G1 exhibited the lowest final microhardness value (*p* < 0.05). Percentage surface microhardness loss (%SHL) analysis confirmed that G2 and G4 experienced significantly lower hardness loss compared to all other experimental groups (*p* < 0.05).

**Table 3:** Mean and standard deviation of KHN before (T0) and after (T1) the erosive–abrasive challenge, and %SHL

Groups	T0 (KHN)	T1 (KHN)	%SHL
	Mean ± SD		
G1 (Negative Control)	352.4 ± 18.6^Aa^	198.3 ± 16.4^Cb^	43.7 ± 5.2^A^
G2 (2.5% *Libidibia ferrea*)	349.8 ± 20.1^Aa^	301.5 ± 15.7^Ab^	13.8 ± 3.8^C^
G3 (0.5% *Libidibia ferrea*)	355.2 ± 19.5^Aa^	235.7 ± 17.1^Bb^	33.6 ± 4.7^B^
G4 (2.5% Brazilian brown propolis)	351.7 ± 17.2^Aa^	309.4 ± 14.8^Ab^	12.0 ± 3.1^C^
G5 (0.5% Brazilian brown propolis)	348.9 ± 16.8^Aa^	241.3 ± 15.5^Bb^	30.8 ± 4.2^B^
G6 (Positive Control)	353.6 ± 18.9^Aa^	252.1 ± 18.6^Bb^	28.7 ± 4.5^B^

Values are presented as mean ± SD (*n* = 12 per group). Different uppercase superscript letters within the same column indicate significant differences between groups. Different lowercase superscript letters within the same row indicate significant differences between T0 and T1 for KHN. KHN was analyzed using two-way mixed-design ANOVA, and %SHL using one-way ANOVA, followed by Tukey’s post hoc comparisons (α = 0.05)

Surface roughness results are presented in Table 4. Baseline Ra values (T0) did not differ significantly among groups (*p* = 0.192). Following the erosive–abrasive challenge (T1), Ra increased significantly in G1, G3, G5, and G6 compared with their respective baseline values (*p* < 0.05). At T1, G2 and G4 exhibited significantly lower Ra values than the other groups (*p* < 0.05).

**Table 4:** Mean and standard deviation of Ra values in µm

Groups	T0 (Ra)	T1 (Ra)
	Mean ± SD	
G1 (Negative Control)	0.15 ± 0.02^Aa^	0.87 ± 0.18^Ab^
G2 (2.5% *Libidibia ferrea*)	0.14 ± 0.01^Aa^	0.18 ± 0.09^Ba^
G3 (0.5% *Libidibia ferrea*)	0.12 ± 0.02^Aa^	0.70 ± 0.16^Cb^
G4 (2.5% Brazilian brown propolis)	0.13 ± 0.04^Aa^	0.16 ± 0.08^Ba^
G5 (0.5% Brazilian brown propolis)	0.15 ± 0.03^Aa^	0.65 ± 0.14^Cb^
G6 (Positive Control)	0.14 ± 0.02^Aa^	0.69 ± 0.12^Cb^

Values are presented as mean ± SD (*n* = 12 per group). Different uppercase superscript letters within the same column indicate significant differences between groups. Different lowercase superscript letters within the same row indicate significant differences between T0 and T1. Data were analyzed using two-way mixed-design ANOVA followed by Tukey’s post hoc comparisons (α = 0.05)

Exploratory analyses using mean values from the four extract-containing gel formulations suggested inverse associations between ABTS radical-scavenging activity and both %SHL and final Ra. Given the small number of formulation-level observations (*n* = 4), these findings are hypothesis-generating and do not establish causality or demonstrate that antioxidant activity mediates the observed enamel outcomes.

### Raman spectroscopy analysis

The full width at half maximum (FWHM) values of the phosphate Raman band at approximately 960 cm⁻¹ are presented in Table 5. Following the erosive–abrasive challenge, G2 and G4 exhibited significantly lower FWHM values than G1, G3, G5, and G6 (*p* < 0.05), with no statistically significant difference between G2 and G4.

**Table 5:** Mean and standard deviation of FWHM expressed as the full width at half maximum of the PO₄³⁻ Raman band at 960 cm⁻¹ after the erosive–abrasive challenge

Groups	FWHM
	Mean ± SD
G1 (Negative Control)	12.1 ± 0.6^A^
G2 (2.5% *Libidibia ferrea*)	6.2 ± 0.2^B^
G3 (0.5% *Libidibia ferrea*)	11.5 ± 0.5^A^
G4 (2.5% Brazilian brown propolis)	5.9 ± 0.2^B^
G5 (0.5% Brazilian brown propolis)	12.3 ± 0.8^A^
G6 (Positive Control)	11.6 ± 0.9^A^

Values are presented as mean ± SD (*n* = 12 per group). Different uppercase superscript letters within the same column indicate significant differences between groups. Data were analyzed using one-way ANOVA followed by Tukey’s post hoc test (α = 0.05)

The phosphate-to-carbonate ratio results are presented in Table 6. Following the erosive–abrasive challenge, G2 and G4 exhibited significantly higher ratios than the other groups (*p* < 0.05). G1 showed the lowest ratio, whereas G3, G5, and G6 presented intermediate values.

**Table 6:** Mean and standard deviation of Phosphate-to-carbonate ratio of enamel specimens following the erosive–abrasive challenge

Groups	PO₄³⁻/CO₃²⁻ ratio
	Mean ± SD
G1 (Negative Control)	6.8 ± 0.9^A^
G2 (2.5% *Libidibia ferrea*)	14.1 ± 1.3^B^
G3 (0.5% *Libidibia ferrea*)	10.0 ± 1.5^C^
G4 (2.5% Brazilian brown propolis)	14.2 ± 1.8^B^
G5 (0.5% Brazilian brown propolis)	10.4 ± 1.1^C^
G6 (Positive Control)	10.5 ± 1.2^C^

Values are presented as mean ± SD (*n* = 12 per group). Different uppercase superscript letters within the same column indicate significant differences between groups. Data were analyzed using one-way ANOVA followed by Tukey’s post hoc test (α = 0.05)

### Color evaluation

Color variation (ΔE00) results are presented in Table 7. G2 and G4 exhibited significantly higher ΔE00 values than the remaining groups (*p* < 0.05), whereas G1, G3, G5, and G6 did not differ significantly from one another (*p* = 0.611). Thus, the 2.5% formulations combined lower %SHL and final Ra values with greater color changes, highlighting a trade-off between the measured enamel outcomes and color alteration.

**Table 7:** Mean and standard deviation of ΔE00 of enamel specimens after the erosive challenge

Groups	ΔE00
	Mean ± SD
G1 (Negative Control)	1.84 ± 0.41^A^
G2 (2.5% *Libidibia ferrea*)	3.72 ± 0.88^B^
G3 (0.5% *Libidibia ferrea*)	1.91 ± 0.45^A^
G4 (2.5% Brazilian brown propolis)	3.68 ± 0.96^B^
G5 (0.5% Brazilian brown propolis)	1.79 ± 0.40^A^
G6 (Positive Control)	1.75 ± 0.42^A^

Values are presented as mean ± SD (*n* = 12 per group). Different uppercase superscript letters within the same column indicate significant differences between groups. Data were analyzed using one-way ANOVA followed by Tukey’s post hoc test (α = 0.05)

## Discussion

The development of bioactive oral gels intended for preventive dental applications requires not only biological efficacy but also adequate physicochemical properties capable of ensuring stability, safety, and clinical applicability. In the present study, all formulations exhibited near-neutral pH values, ranging from 6.91 to 7.43, which is an important characteristic considering that acidic formulations may contribute to enamel demineralization and compromise long-term biocompatibility. Furthermore, the incorporation of *Libidibia ferrea* and Brazilian propolis extracts resulted in marked differences in total phenolic content and antioxidant activity, with the 2.5% *Libidibia ferrea* gel showing the highest concentration of extractable polyphenols and the greatest radical-scavenging capacity. These findings are consistent with previous reports demonstrating that *Libidibia ferrea* fruits are particularly rich in hydrolysable tannins, gallic acid derivatives, flavonoids, and other phenolic compounds that contribute substantially to their antioxidant potential [19, 20]. Similarly, propolis contains a complex mixture of phenolic acids, flavonoids, and prenylated compounds recognized for their antioxidant and biomodulatory activities [21, 23]. Therefore, the chemical characterization confirmed the successful incorporation of bioactive compounds into the gel matrix.

From a pharmaceutical perspective, the rheological and stability analyses demonstrated that the incorporation of natural extracts did not adversely affect the physical behavior of the Carbopol-based system. All formulations exhibited pronounced shear-thinning behavior, characterized by a progressive reduction in viscosity as the shear rate increased. This pseudoplastic profile is highly desirable for oral gels because it facilitates product spreading during application while maintaining sufficient viscosity at rest to prolong contact with the tooth surface and oral tissues. Similar rheological behavior has been reported for Carbopol-containing oral formulations and is generally associated with improved retention and patient acceptability [28, 35]. The formulations remained stable throughout refrigerated storage and during the alternating-temperature cycles. However, color changes and precipitate formation were observed at room temperature after 90 days and at elevated temperature from day 15 onward, indicating storage-dependent stability limitations. The color changes and precipitate formation detected under accelerated aging conditions are likely associated with oxidation and polymerization reactions involving phenolic compounds, phenomena frequently reported in plant-derived formulations rich in tannins and flavonoids [36]. Collectively, these findings indicate that the experimental gels possess suitable physicochemical properties for oral application and provide an appropriate vehicle for delivering polyphenol-rich bioactive compounds to dental surfaces.

In addition to presenting suitable physicochemical characteristics, all experimental formulations exhibited satisfactory cytocompatibility toward human gingival fibroblasts, with cell viability values consistently above the 70% threshold established by ISO 10993-5 for classification as non-cytotoxic materials. Although cell viability was lower at the higher extract concentrations, particularly for the 2.5% *Libidibia ferrea* gel, all formulations remained above the 70% viability threshold under the tested conditions. These findings are in agreement with previous investigations reporting low cytotoxicity and favorable biocompatibility profiles for both *Libidibia ferrea* extracts and propolis-derived products. Phenolic compounds, flavonoids, and tannins present in these natural products have been shown to exert antioxidant and anti-inflammatory effects while maintaining cellular viability at concentrations compatible with biomedical applications [19, 23, 24]. From a translational perspective, these findings are particularly relevant because oral gels are intended for prolonged contact with oral soft tissues, making biocompatibility a fundamental prerequisite for future clinical application.

Microhardness analysis indicated concentration-dependent effects under the experimental erosive–abrasive conditions. Although enamel hardness decreased in all groups, the 2.5% *Libidibia ferrea* and Brazilian brown propolis gels exhibited significantly lower %SHL and higher final Knoop hardness values than the other treatments, including the commercial stannous fluoride toothpaste. These findings do not establish superior clinical efficacy or a concentration threshold for clinically relevant protection. Differences in delivery vehicle and product composition also limit direct comparisons of the active ingredients. Surface microhardness is a sensitive indicator of enamel surface softening during the early stages of erosion [37, 38]. Previous studies suggest that polyphenols may adsorb onto dental surfaces and interact with salivary proteins, potentially modifying surface properties and limiting acid diffusion and mineral dissolution [10]. Such processes could help explain the lower %SHL observed with the 2.5% formulations. However, polyphenol adsorption, pellicle modification, and hydrogen ion diffusion were not directly assessed, and their contribution remains hypothetical.

The surface roughness findings corroborated the microhardness results and further reinforced the protective effect of the 2.5% formulations against erosive–abrasive wear. Whereas the control gel, the lower-concentration formulations, and the stannous fluoride group exhibited significant increases in roughness after the experimental challenge, the 2.5% *Libidibia ferrea* and 2.5% propolis gels-maintained surface characteristics closer to baseline conditions. Increased roughness is a hallmark of erosive enamel dissolution and reflects the preferential removal of interprismatic mineral, resulting in a more irregular and porous surface topography [4]. Therefore, the lower roughness values observed in the highly concentrated formulations suggest a reduced degree of mineral dissolution and surface degradation. These findings are clinically relevant because rougher enamel surfaces exhibit greater plaque accumulation, increased susceptibility to staining, and enhanced progression of erosive wear over time [39]. The lower roughness values observed for the 2.5% formulations may be partially explained by the interaction of polyphenolic compounds with the enamel surface and salivary proteins. Niemeyer et al. (2023) [40] demonstrated that plant extracts rich in polyphenols protect dental tissues through a dual mechanism involving direct modification of the substrate and alterations in the acquired salivary pellicle, increasing its resistance to erosive attacks. Although the experimental model included a human salivary pellicle, its presence does not establish that the formulations modified its composition or protective properties. Pellicle modification is therefore a possible explanation based on previous literature, rather than a mechanism demonstrated by the present findings.

Exploratory regression analyses suggested that formulations with greater ABTS radical-scavenging activity tended to exhibit lower %SHL and final Ra values following the erosive–abrasive challenge. These associations were based on only four formulation-level observations and therefore remain hypothesis-generating. They do not demonstrate that antioxidant activity caused the observed effects, as other compositional differences among formulations may also contribute to these relationships. Previous studies have reported that plant-derived extracts can reduce erosive damage through interactions with the dental substrate and salivary pellicle [40, 41]. However, those findings do not establish antioxidant activity as the mechanism responsible for the enamel outcomes observed here.

Furthermore, previous studies have suggested that polyphenolic compounds may interact with the mineral phase of dental tissues, potentially contributing to greater resistance to erosive challenges. Such interactions have been proposed as one of the mechanisms by which polyphenol-rich products help preserve dental hard tissues exposed to acidic environments [42]. The higher polyphenol content of the 2.5% formulations offers a possible explanation for their lower %SHL and final Ra values. However, pellicle modifications and substrate–polyphenol interactions were not directly assessed. The exploratory formulation-level associations therefore remain hypothesis-generating and do not establish which compounds or mechanisms contributed to the observed enamel outcomes.

The Raman spectroscopy findings provided complementary information on enamel mineral parameters following the erosive–abrasive challenge. The 2.5% *Libidibia ferrea* and Brazilian brown propolis gels exhibited significantly lower FWHM values for the ν₁ phosphate band than the remaining groups. The full width at half maximum of this band is commonly used as an indirect indicator of mineral crystallinity in dental tissues, with lower values being consistent with a more organized apatite structure [43, 44]. Accordingly, the differences observed suggest greater crystal organization in specimens treated with the 2.5% formulations at the post-challenge assessment. However, these measurements alone do not establish that the formulations preserved crystallinity throughout cycling. Although the Raman findings accompanied lower microhardness loss and final roughness values, this combination does not directly demonstrate preservation of enamel structural integrity.

The phosphate-to-carbonate ratio complemented the FWHM analysis by providing information on the relative mineral contributions to the Raman spectra after the erosive–abrasive challenge. The highest ratios were observed for the 2.5% *Libidibia ferrea* and Brazilian brown propolis gels, whereas the control gel group exhibited the lowest value. Carbonate substitution is a common feature of biological apatites and can increase crystal solubility, influencing their susceptibility to acidic dissolution [37, 38]. This relationship provides a basis for considering the observed ratios when interpreting enamel mineral characteristics. Nevertheless, a higher phosphate-to-carbonate ratio does not directly quantify mineral loss or establish reduced demineralization. Together, the ratio and FWHM findings indicate differences in post-challenge mineral parameters among treatments, while the processes responsible for those differences remain undetermined.

The magnitude of the Raman differences was substantial: FWHM values for the 2.5% formulations were approximately half those observed in the control gel and 0.5% formulation groups, while their phosphate-to-carbonate ratios were approximately twice that of the control gel group. These contrasts highlight the differences detected under the present experimental conditions, although caution is warranted when comparing absolute Raman values across studies. Their occurrence alongside lower %SHL and final Ra values provides complementary evidence of treatment-related differences across the measured enamel outcomes. However, the magnitude and consistency of these findings do not establish preservation of the enamel mineral phase or identify a common protective mechanism. Further investigations directly assessing mineral loss and surface chemical interactions would help clarify their biological significance.

Despite their superior protective performance against erosive–abrasive wear, the 2.5% *Libidibia ferrea* and 2.5% propolis gels exhibited significantly greater color changes than the other experimental groups. This finding was expected, considering the high concentration of naturally pigmented phenolic compounds present in both extracts. Polyphenols, tannins, and flavonoids are known to possess chromophoric structures capable of interacting with dental surfaces and contributing to color alterations, particularly when present at higher concentrations [42]. Although the present study was not designed to investigate the mechanisms responsible for these color changes, the results suggest that increasing extract concentration may influence the esthetic properties of the treated enamel. Therefore, future studies should evaluate whether formulation adjustments, alternative concentrations, or modified delivery systems could preserve the anti-erosive benefits of these compounds while minimizing undesirable color alterations.

The concentration-dependent findings highlight considerations for further formulation development. Although the 2.5% gels exhibited lower %SHL and final Ra values, they also produced greater color changes. The smaller color changes observed with the 0.5% formulations suggest that extract concentration may influence the balance between enamel outcomes and esthetic effects, but do not establish patient acceptability. The stability limitations observed at higher concentrations must also be considered when assessing their potential application. Further optimization should therefore address extract concentration, color alteration, and storage stability before clinical evaluation.

Some limitations should be considered when interpreting the present findings. The mechanisms underlying the observed enamel outcomes were not directly investigated; proposed explanations involving polyphenol-rich extracts therefore rely on previous literature rather than direct mechanistic evidence. Furthermore, the exploratory regression analyses were based on only four formulation-level observations, and the associations between antioxidant activity and enamel outcomes remain hypothesis-generating rather than confirmatory. The in vitro design does not establish long-term clinical efficacy, safety, or acceptability. Cytocompatibility in the tested cell model provides preliminary biological information rather than confirmation of clinical safety, while the observed color changes and storage instability require further evaluation. These findings support continued formulation development but do not yet justify recommendations for clinical use. Future in situ and clinical studies, complemented by surface chemical analyses and characterization of the acquired salivary pellicle, are needed to investigate the proposed mechanisms and evaluate efficacy, safety, and acceptability under clinically relevant conditions.

## Conclusion

Within the limitations of this in vitro study, bioactive oral gels containing *Libidibia ferrea* and Brazilian propolis demonstrated promising protective effects against enamel erosive–abrasive wear. The formulations exhibited suitable physicochemical properties, satisfactory cytocompatibility, and more favorable balance enamel mechanical and mineral characteristics following the erosive–abrasive challenge. Under the present experimental conditions, the 2.5% formulations showed lower microhardness loss and surface roughness, together with lower phosphate ν₁ band FWHM values and higher phosphate-to-carbonate ratios. However, these formulations were also associated with greater color alteration, whereas the 0.5% formulations offered a more favorable balance between protective efficacy and esthetic outcomes. Overall, the findings support the potential of polyphenol-rich oral gels as adjunctive strategies for the prevention of erosive tooth wear and provide a basis for future in situ and clinical investigations.

## Data Availability

The raw data generated and analyzed during the current study are available from the corresponding author upon reasonable request.
